# Assessing Cognitive Function and Grip Strength in Chronically Homeless Individuals

**DOI:** 10.1002/alz70860_101371

**Published:** 2025-12-23

**Authors:** Gesulla Cavanaugh, Stachyse Stanis, Raina Patel

**Affiliations:** ^1^ Nova Southeastern University, Ft Lauderdale, FL, USA

## Abstract

**Background:**

Individuals living in poverty may experience premature cognitive impairment and premature aging, which are associated with biological deterioration, and may lead to early onset of dementia.

**Method:**

Adults experiencing homelessness (*N* = 60) for at least 12 months and non‐homeless (*N* = 45) participated in this study. Validated assessment tools along with biomedical analyses were utilized to provide a thorough evaluation of the targeted groups’ cognitive health, health literacy, and biological and physical health. Cognitive function was assessed using the Montreal Cognitive Assessment (MoCA), the Comprehensive Trail Making Test, and the Hopkins Verbal Learning Test. Cognitive load was evaluated through pupil diameter captured with the Tobii Pro Nano eye tracking device, while grip strength was measured with a handheld dynamometer, and biological health was assessed through telomere length analysis.

**Result:**

Individuals from the homeless group scored significantly lower in the HVLT and the C‐TMT; similarly, the mean MoCA score was higher in the non‐homeless group than in the homeless group (26.4 ± 2.07 and 20.1 ± 2.86, *p* = 0.000) with evidence of higher increase in cognitive load during a health literacy test in the homeless group. On average, the homeless group spent significantly longer time completing the health literacy assessment and the cognitive tests, suggesting correlations with processing speed, executive functioning and memory. The homeless group's MoCA and Hopkins Verbal Learning test scores were consistent with weaker grip strength (M = 31.3kg ±11.6 kg vs 40kg ±7.8kg), pointing to a correlation between cognitive weakening and frailty.

**Conclusion:**

This pilot study focuses on the association between psychosocial stress and accelerated aging, particularly in the homeless person. The results suggest that individuals exposed to chronic stressors may experience premature aging and premature cognitive decline, which may impact cognitive function.

